# Methylene Blue Penetration of Resin Infiltration and Resin Sealant in Artificial White-Spot Lesions

**DOI:** 10.1055/s-0042-1756689

**Published:** 2022-10-11

**Authors:** Awiruth Klaisiri, Jarukit Vongsang, Thanach leelaudom, Nantawan Krajangta

**Affiliations:** 1 Division of Restorative Dentistry, Faculty of Dentistry, Thammasat University, Pathumthani, Thailand; 2 Thammasat University Research Unit in Restorative and Esthetic Dentistry, Thammasat University, Pathumthani, Thailand; 3 Faculty of Dentistry, Thammasat University, Pathumthani, Thailand

**Keywords:** methylene blue, microleakage, resin infiltration, resin sealant, white-spot lesion

## Abstract

**Objective** This study determined the potency of resin infiltrations and resin sealant in impeding microleakage on artificial white-spot lesions (AWL) by methylene blue penetration.

**Materials and Methods** Eighty AWL specimens were randomly separated into two groups for water storage for 24 hours (groups 1–4) and 20,000 cycles of thermocycling (TC) (groups 5–8). Each group was then separated into four subgroups ( *n* = 10) based on the AWL surface treatments: (1) no Tx, (2) treated with resin infiltration (ICON, DMG, Hamburg, Germany), (3) treated with resin infiltration (Surface pre-reacted glass-ionomer (S-PRG) barrier coat, SHOFU, Kyoto, Japan), (4) treated with resin sealant (Clinpro sealant, 3M ESPE, Minnesota, United States). Nail varnish was covered to all samples, with the exception of a 4 × 4 mm ^2^ patch on the buccal measurement region, which was subsequently submerged in a 2% methylene blue solution and cut into buccolingual sections. Stereomicroscope measurements were used to calculate methylene blue penetration scores.

**Statistical Analysis** The Kruskal–Wallis test and the Bonferroni post-hoc correction were performed to evaluate the data.

**Results** Application of resin infiltrants and resin sealant reduced microleakage in AWL by methylene penetration both before and after thermal cycling. In addition, group 6 (ICON + TC) and group 7 (S-PRG + TC) had a significantly different value from group 8 (sealant + TC).

**Conclusion** Both the resin infiltration approach and the resin sealant seem to help seal AWL and might perhaps offer long-term defense against microleakage in AWL caused by methylene blue penetration. The greatest sealing and defense for microleakage in AWL were demonstrated by the resin infiltrations.

## Introduction

One of the really frequent diseases of the tooth is dental caries. It is caused by an imbalance within demineralization and remineralization processes throughout time. A white-spot lesion or initial carious lesion is the earliest clinical marker of enamel carious lesions. When they are on a smooth surface, they look as chalky white lesions that are microporous and rough. 1 After a 1-year follow-up, initial carious lesions healed in 57.1% of cases without therapy. 2 Nevertheless, initial carious lesions could not be totally eliminated, according to Al-Khateeb et al. 3 Thus, it is important to stop these lesions from progressing, which is the first barrier before cavitation. Because they conserve the natural tooth structure, improve clinical results, and promote esthetics in restorative and conservative treatments, minimally invasive treatments are an essential strategy for managing white-spot lesions.

Resin sealants can protect and inhibit the progress of initial caries through creating as a barricade with the oral cavity environment and the tooth surface. 4 5 6 Prior research revealed that resin sealants efficiently prevent tooth decay, although their longevity is debatable. 7 8 Therefore, microleakage surrounding the resin sealant decreases its efficacy if any part of it is displaced from its site. 9 Resin sealants show an 80% retention rate at 2 years, a 70% retention rate at 4.5 years, and a 39% retention rate at 9 years, according to Bravo et al. 10

Resin infiltration is a novel access in the field of minimally invasive dentistry. Resin infiltration stopped enamel demineralization and corrected the appearance of white-spot lesions. 11 12 13 This procedure seems to be microinvasive; therefore, it could act as a bridge between noninvasive and minimally invasive procedures for treating initial carious lesions. 14 The goal of this approach is to use resin monomer to infill the intercrystalline micropores inside the lesion body. According to Enan et al, 15 resin infiltrant improved demineralized enamel surface tolerance to acidic attack.

Clinical success is significantly influenced by a resin monomer's ability to decrease microleakage at the restoration and the tooth interface. 16 17 18 To determine leakage *in vitro* , organic dyes are often used. Because the molecular size of methylene blue dye (0.5–0.7 nm) is lower than that of bacteria, it was applied in this investigation to evaluate resin infiltrant leakage and the tooth interface. 19 Few investigations have explored the influence of resin infiltrant and resin sealant on initial carious lesion microleakage. Therefore, the purpose of this research was to determine the performance of resin infiltration and resin sealant in preventing microleakage on artificial white-spot lesions (AWL) by methylene blue penetration.

## Materials and Methods

### Preparation of Specimens

Eighty normal premolar teeth were prepared and stored in a 0.1% thymol solution for no more than 30 days before the experiment was investigated. The ethical experimental study received approval from the ethics subcommittee for human research in the sciences of Thammasat University (ECScTU): COE number 028/2563.

### AWL Formation

The specimen was submerged in demineralizing and remineralizing solutions to form AWL. Mix 0.9 mM of KH _2_ PO _4_ , 1.5 mM of CaCl _2_ , and 50 mM of acetic acid to produce the demineralizing solution. The demineralizing solution pH was corrected with 1 M of KOH to 5.0. The pH meter (GSS-304B, DKK-TOA Corporation, Tokyo, Japan) was used to check the pH value. The samples were incubated in the demineralizing solution for 14 days at 37°C, with the solution being changed every day. After that, the specimens were removed and properly washed for 1 minute with distilled water. Demineralized teeth were treated in a remineralizing solution comprising 0.9 mM of KH _2_ PO _4_ , 1.5 mM of CaCl _2,_ 20 mM of 4-(2-hydroxyethyl)-1-piperazineethanesulfonic acid, and 130 of mM KCl to create an initial carious lesion's surface layer. The remineralizing solution pH was corrected with 1 M of KOH to 7.0. The demineralized teeth were incubated in the remineralizing solution for 14 days at 37°C, with the solution being changed every day. After removing the specimens from the remineralizing solution, they were washed for 1 minute with distilled water. 11 18

The AWL samples were randomly split into two groups for water storage for 24 hours (groups 1–4) and 20,000 cycles of thermocycling (groups 5–8) and each group was then separated into four subgroups ( *n* = 10) based on the surface treatments applied to the AWLs: (1). no Tx; (2) treated with resin infiltration (ICON, DMG, Hamburg, Germany); (3) treated with resin infiltration (Surface pre-reacted glass-ionomer (S-PRG) barrier coat, SHOFU, Kyoto, Japan); (4) treated with resin sealant (Clinpro sealant, 3M ESPE, Minnesota, United States).

### Application of Resin Infiltration or Resin Sealant

Resin infiltration (ICON) was performed on the specimens' buccal surface. Pumice was used to clean the samples, followed by distilled water washing, 2 minutes of etching using 15% hydrochloric acid (HCl), a thorough rinse, and 30 seconds of air-water drying. A thirty-second application of ICON-dry was followed by a five-second drying. Resin infiltrant was treated, left approximately 3 minutes and light-cured for 40 seconds (Demi Plus dental curing light, Kerr Corporation, California, United States) and then reapplied and left approximately 1 minute, and given a 40-second light cure.

Resin infiltration (S-PRG barrier coat) was performed on the specimens' buccal surface. The samples were scrubbed with pumice, washed with distilled water, and then dried. Base and activator of S-PRG barrier coat were combined with a microbrush, and then applied on the specimens' buccal surface, left approximately 5 seconds, given a 20-second light cure, and a cotton pellet was used to cleanse the coating's surface.

Resin sealant (Clinpro sealant) was performed on the specimens' buccal surface. After being cleaned using pumice, thoroughly rinsed, etched for 15 seconds using 37% phosphoric acid, and then completely rinsed and dried approximately 30 seconds with an air-water spray. The resin sealant was treated and given a 20-second light cure.

Table 1 shows the materials used in this study.

**Table 1:** Composition of resin sealant and resin infiltration

Material	Composition
Resin sealant; Clinpro sealant (3M ESPE, Minnesota, United States) Lot number: NF15372	Bis-GMA, TEGDMA, tetrabutylammonium tetrafluoroborate, dichloride methylsilane, silica, titanium dioxide
Resin infiltration; S-PRG barrier coat (SHOFU, Kyoto, Japan) Lot number: 051801	Base: S-PRG filler, methacrylic acid monomer, distilled water, others Active: Phosphonic acid monomer, carboxylic monomer, methacrylic acid monomer, TEGDMA, Bis-MPEPP, initiator, others
Resin infiltration; ICON (DMG, Hamburg, Germany **)** Lot number: 733275	Etch: 15% hydrochloric acid Dry: 99% ethanol Infiltrant: TEGDMA-based resin, initiators, stabilizers

**Abbreviations:** Bis-GMA, bisphenol A-glycidyl methacrylate; Bis-MPEPP, 2,2'-bis(4-methacryloxy polyethoxyphenyl) propane; S-PRG, surface pre-reacted glass-ionomer; TEGDMA, triethylene glycol dimethacrylate.

### Thermocycling Procedure

The specimens were preserved in an incubator at a temperature of 37°C and a humidity of 100% for 24 hours (Contherm, Contherm Scientific Ltd., Lower Hutt, New Zealand). Then, 20,000 thermocycles were performed on the samples of groups 5 to 8. Thermocycling was performed using bath temperatures of 5 and 55°C, a 30-second dwell period in each bath, and a 5-second transfer period.

### Microleakage of AWL by Methylene Blue Penetration

Nail varnish was covered to all samples, with the exception of a 4 × 4 mm ^2^ area here on buccal surface that was designed to evaluate microleakage. The samples were then submerged in a 2% of methylene blue solution for 24 hours at 37°C (immersion period followed by ISO/TS 11405). 20 After thoroughly cleaning each specimen under running water, a slow cutting device was used to segment each specimen buccolingually (Isomet, Buehler Ltd., Illinois, United States). A stereomicroscope with a 50x magnification was used to examine the sectioned samples (ML9300, Meiji Techno Co. Ltd., Saitama, Japan). By measuring methylene blue penetration, microleakage was graded as follows. 18

0 = no methylene blue penetration
1 = the outer half of the enamel is penetrated by methylene blue
2 = the inner half of the enamel is penetrated by methylene blue
3 = the outer half of the dentin is penetrated by methylene blue
4 = the inner half of the dentin is penetrated by methylene blue

### Statistical Analysis

Statistical software was used to compare methylene blue penetration scores between groups using the Kruskal–Wallis test and post-hoc Bonferroni adjustment for pairwise comparison at a 95% level of confidence. The cutoff for significance was chosen at *p* < 0.05.

## Results

**Fig. 1:**
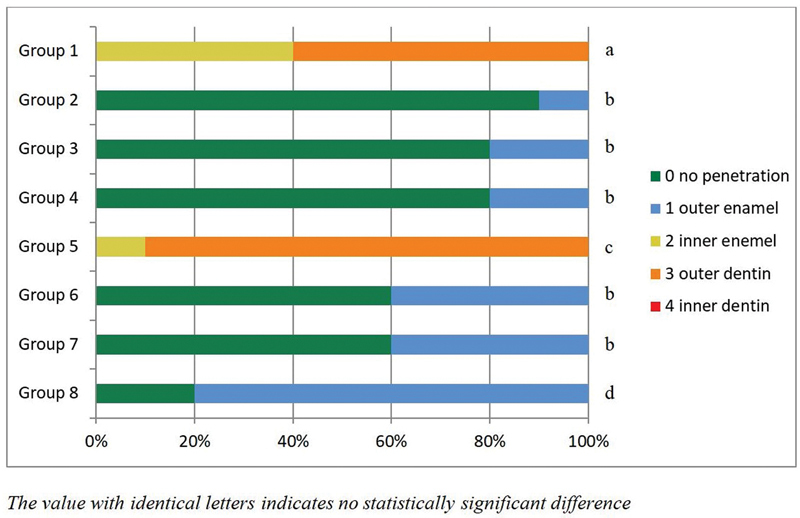
Percentage of methylene blue penetration dye.

In this investigation, resin infiltration and resin sealant inhibited methy-lene blue penetration. Table 2 shows methylene blue penetration scores and Fig. 1 shows percentage of methylene blue penetration in all groups. In group 1, methylene blue penetrated to the enamel's inner layer at 40% and the dentin's outer layer at 60%. Groups 2, 3, and 4's values did not significantly differ from one other. In aging process, methylene blue penetrated to the enamel's inner layer at 10% and the dentin's outer layer at 90% in group 5. The values for group 6 and group 7 were significantly different from group 8.

**Table 2:** The methylene blue penetration scores

Groups			Scores		
	0	1	2	3	4
1. No Tx	0	0	4	6	0
2. ICON	9	1	0	0	0
3. S-PRG	8	2	0	0	0
4. Sealant	8	2	0	0	0
5. No Tx + TC	0	0	1	9	0
6. ICON + TC	6	4	0	0	0
7. S-PRG + TC	6	4	0	0	0
8. Sealant + TC	2	8	0	0	0

## Discussion

The *in vitro* assessment of microleakage by methylene blue penetration of resin infiltration and resin sealant currently lacks a standardized methodology, making it challenging to compare the findings of different investigations. 18 21 22 We defined microleakage as the methylene blue penetration along the tooth and the resin material interface. The methylene blue penetration test gives important information to determine the microleakage of restorative materials. However, microleakage in the oral environment, which can be caused by several reasons, is still a problem. So, this investigation determined the potency of resin infiltrations and resin sealant in impeding microleakage on AWL by methylene blue penetration. Our study showed both the resin infiltration approach and the resin sealant seem to help seal AWL and might perhaps offer long-term defense against microleakage in AWL caused by methylene blue penetration. Moreover, the ICON and S-PRG barrier coat showed superior sealing and protection for microleakage in AWL compared with the resin sealant after the thermal cycling process.

The evaluation of the deterioration of restorative materials was made possible via thermocycling, which was employed to produce a simulated aging process. 23 The failure of the material is caused by stress that is created by the thermal contraction and expansion of the resin material in relation to the tooth. 24 Intermittently using hot and cold water can cause exposed collagen fibrils to hydrolyze caused by repeated contraction and expansion stress, which causes microleakage to develop along the resin material interface. 25 For our study, 20,000 cycles of thermocycling were used to simulate oral aging. 26

The resin sealant, Clinpro sealant, had to be etched with 37% phosphoric acid, washed, dried, and then applied and allowed to cure. The decreased viscosity of Clinpro sealant allowed for better penetration in tight places and a stronger bonding in the enamel's deep layers. 27 According to Nahvi et al, the etch and rinse resin sealant has higher microleakage by methylene blue penetration compared with the self-etch resin sealant. 9 In the present study, Clinpro sealant showed the best sealing in the prior aging process and decreased performance for microleakage in AWL after the thermal cycling process. This is due to sealants' larger thermal expansion coefficient than enamel's. As a result, gaps are created in the oral cavity due to the ongoing temperature variations, which make it easier for methylene blue to penetrate the sealant and enamel interface. 28

The resin infiltration, S-PRG barrier coat, was established for their constructional and ion-releasing mechanisms. This material may release a variety of ions, such F, Si, Al, Na, B, and Sr ions. It also contains a self-etching bioactive base and active liquids. 29 Water will be absorbed by the S-PRG barrier coat and released along with the ions, but the filler will not degrade. Due to their stability over a long period of time in contact with saliva, these fillers can be employed in the creation of resin materials. 30 Sealing the AWL with a resinous layer that self-etches and contains inorganic ion fillers as S-PRG barrier coat is one of the most crucial steps. To prevent methylene blue from penetrating, this coating layer can serve as a barrier. 29 Moreover, through the transformation of hydroxyapatite into fluorapatite and strontium apatite, fluoride and strontium also help teeth be more resistant to acid. 31 In the present investigation, S-PRG barrier coat showed the best sealing and protection for microleakage in AWL by methylene blue penetration both of before and after aging process. These methods listed above are the most likely causes for the S-PRG barrier coat's ability to protect from microleakage in AWL caused on by methylene blue penetration.

The resin infiltration, ICON, uses a low-viscosity resin monomer to aim to penetrate enamel white-spot lesions. For the adaptability and longevity of resin infiltration, triethylene glycol dimethacrylate (TEGDMA) penetration of the microporosities produced by the hydrochloric acid (HCl) etchant is required. 32 HCl has been shown to be a far more destructive etchant than a 37% phosphoric acid etchant. 33 Therefore, this destructive may have caused ICON to be penetrated deeper and sealed as a result. According to the current findings, there was a significant difference in the methylene blue infiltration depth prior to thermocycling between specimens treated with resin infiltration and those that were not. Similar to this, Lee et al discovered that the methylene blue penetration depth was much lower for enamel lesions with resin infiltrated surfaces compared with nontreated enamel. 34 Moreover, ICON has a greater capacity for penetration and a tooth-like coefficient of thermal contraction and expansion. 22 According to Klaisiri et al, white-spot lesions in enamel are promptly sealed and protected from microleakage by TEGDMA in the aging process. 18 In our study, ICON demonstrated the best sealing and protection from microleakage in AWL by methylene blue penetration both prior to and after 20,000 cycles of thermocycling.

To the best of the research findings, this study determined the effectiveness of resin infiltrations (ICON and S-PRG barrier coat) and resin sealant (Clinpro sealant) in inhibiting microleakage on AWL by methylene blue penetration. Nevertheless, there are some experimental research limitations. First, the design of the *in vitro* experimental investigation endangers the generalizability or external validity of the findings in clinical dental application. Second, there may be variations between lots of resin infiltrations and resin sealant. Standardization of manufacturing is above the purview of this investigation. Finally, the clinical success of materials is influenced by several factors, not just microleakage. Therefore, our research's findings should be interpreted cautiously.

## Conclusion

Under our research situations, both the resin infiltration approach and the resin sealant seem to help seal AWL and might perhaps offer long-term defense against microleakage in AWL caused by methylene blue penetration. The best sealing and defense from microleakage in AWL after the aging process were demonstrated by the resin infiltrations (ICON and S-PRG barrier coat).
